# Genome Sequence of Komagataeibacter saccharivorans Strain JH1, Isolated from Fruit Flies

**DOI:** 10.1128/MRA.00098-20

**Published:** 2020-03-26

**Authors:** Jacqueline Hollensteiner, Anja Poehlein, Patrick Kloskowski, Tariq Tammam Ali, Rolf Daniel

**Affiliations:** a Department of Genomic and Applied Microbiology, Institute of Microbiology and Genetics, University of Göttingen, Göttingen, Germany; b Göttingen Genomics Laboratory, Institute of Microbiology and Genetics, University of Göttingen, Göttingen, Germany; Georgia Institute of Technology

## Abstract

We present the genome sequence of Komagataeibacter saccharivorans strain JH1, which belongs to the acetic acid bacteria. The draft genome sequence consists of 3.7 Mb and contains 3,437 predicted protein-encoding genes. This organism contains putative genes involved in cellulose and levan biosynthesis.

## ANNOUNCEMENT

Komagataeibacter saccharivorans strain JH1 belongs to the genus *Komagataeibacter* of the acetic acid bacteria (1). Members of this genus are known to produce exopolysaccharides such as cellulose (2), which are of biotechnological importance (3).

K. saccharivorans JH1 was originally isolated from fruit flies collected in Göttingen, Germany, in May 2017. The flies were used as starters for enrichment cultures of acetic acid bacteria, containing 50 ml nonsulfated white wine, 50 ml sterile water, and 0.1% cycloheximide. Cultures were incubated at 28°C for 7 days. Subsequently, single colonies of potential acetic acid bacteria were isolated by streaking onto glucose-yeast-peptone (GYP) indicator medium (4). Acid producers were restreaked and incubated at 28°C for 3 days on GYP medium (DSMZ medium 1295; Deutsche Sammlung für Mikroorganismen und Zellkulturen, Braunschweig, Germany) and were checked via 16S rRNA gene colony PCR (5) and Sanger sequencing (SeqLab, Göttingen, Germany). The recovered 16S rRNA gene sequences revealed a sequence identity of 99% to the 16S rRNA gene sequences of other K. saccharivorans strains. For genome sequencing, colony material was used to inoculate 5 ml GYP medium. After growth at 28°C for 4 days, cells were harvested by centrifugation, and genomic DNA was extracted by using the MasterPure complete DNA purification kit as recommended by the manufacturer (Epicentre, Madison, WI, USA). Illumina paired-end shotgun libraries were prepared by using the Nextera XT DNA sample preparation kit and were sequenced by employing the MiSeq system and reagent kit v3 (2 × 300 bp) as recommended by the manufacturer (Illumina, San Diego, CA, USA). For Nanopore sequencing, 1.5 μg DNA was used for library preparation employing the ligation sequencing kit 1D (SQK-LSK108) and the native barcode expansion kit (EXP-NBD103, barcode 9) as recommended by the manufacturer (Oxford Nanopore Technologies, Oxford, UK). Sequencing was performed using a MinION Mk1B device and a SpotON R9.4 flow cell, as recommended by the manufacturer (Oxford Nanopore Technologies). MinKNOW software v15.1.1 was employed for sequencing, and Albacore v2.3.1 was used for demultiplexing. Default parameters were used for all software unless otherwise specified. Quality filtering of the reads using fastp v0.19.4 (6) resulted in 51,817 Nanopore reads (mean read length, 1,620 bp) and 2,746,722 Illumina reads. Unicycler v0.4.4 (7) was used for hybrid assembly, which yielded 15 contigs (>500 bp) and 243.6-fold coverage, as validated by Bandage v2.1 (8). The genome sequence consists of 3,727,857 bp, with an overall GC content of 61.3%, including 1 closed circular chromosome (3,105,849 bp) and 5 closed plasmids (ranging from 3 to 222 kb). Based on bridging and coverage with Bandage v2.1 (8), 1 unclosed plasmid with 9 contigs (ranging from 532 to 183,533 bp) remained.

**FIG 1:**
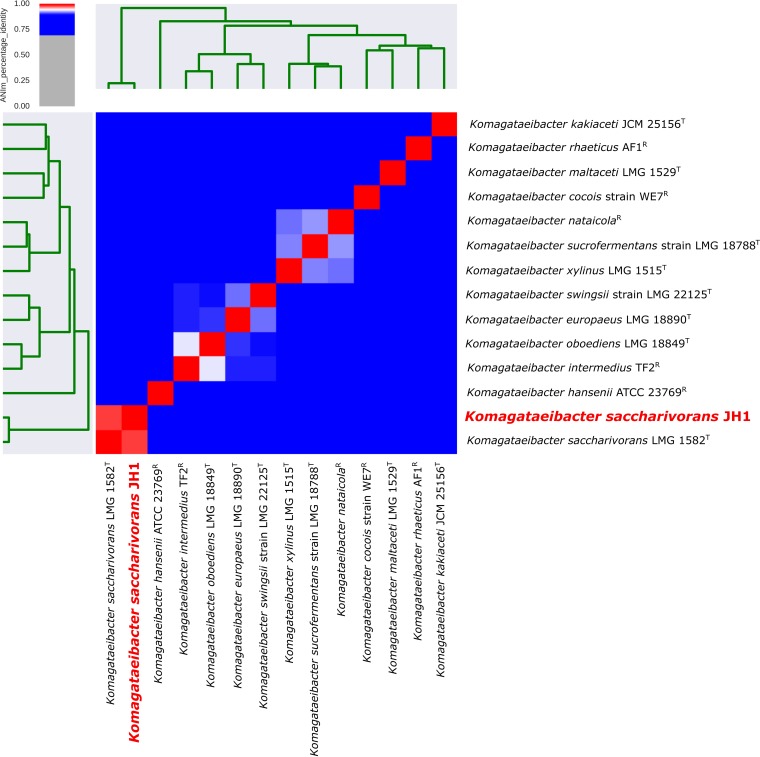
Phylogenetic analysis of K. saccharivorans JH1 (red). Genome sequences for all available type strains (T) and representative strains (R) from the genus *Komagataeibacter* were taken into account. Calculations were performed with PyANI (10) using the MUMmer average nucleotide identity (ANIm) method with standard parameters, which revealed a sequence identity of 98.5% between K. saccharivorans JH1 and the type strain K. saccharivorans LMG1582, which is higher than the species boundary of approximately 94%.

Annotation performed using the Prokka tool v1.13.3 (9) predicted 3,512 genes, including 59 tRNA genes, 1 transfer mRNA gene, and 3,437 protein-encoding genes, of which 1,646 had predicated functions. Phylogenetic analysis using PyANI v0.2.7 (10) showed that K. saccharivorans JH1 exhibited 98.7% sequence identity to the type strain K. saccharivorans LMG 1582 (GenBank accession number NKTY00000000) (Fig. 1).

Using BlastKOALA v2.1 (11), the genome revealed the presence of putative genes encoding a cellulose synthase (KSAC_25330, KSAC_25280, and KSAC_25340), a levansucrase (KSAC_01950), and a levanase (KSAC_01960). Thus, the ability of K. saccharivorans JH1 to perform cellulose and levan biosynthesis is indicated.

### Data availability.

This whole-genome shotgun project has been deposited at DDBJ/ENA/GenBank under accession numbers CP036404 (chromosome) and CP036405 to CP036418 (plasmids). The NCBI BioProject accession number is PRJNA523114, and the raw reads have been deposited in the NCBI SRA database under accession number SRP200000.
